# Do we have to reduce the recall period? Validity of a daily physical activity questionnaire (PAQ24) in young active adults

**DOI:** 10.1186/s12889-020-8165-3

**Published:** 2020-01-16

**Authors:** B. Novak, P. Holler, J. Jaunig, W. Ruf, M. N. M. van Poppel, M. C. Sattler

**Affiliations:** 1 Institute of Sport Science, University of Graz, Graz, Austria; 2 Department of Public Health, Institute for Health Promotion and Disease Prevention (IfGP), Graz, Austria; 3 Institute of Sport Science, German University of Health and Sports, Berlin, Germany

**Keywords:** Physical activity, Questionnaire, Self-report, Measurement, Validity

## Abstract

**Background:**

Combining the strengths of physical activity (PA) diaries and questionnaires may be needed to improve the unsatisfying measurement quality of existing PA questionnaires. This study investigated the construct validity of a short PA questionnaire (Physical Activity Questionnaire for 24 h [PAQ24]) with a recall period of one day.

**Methods:**

In this cross-sectional study, participants completed the PAQ24 on seven consecutive days while wearing an accelerometer (GENEActiv). Thereafter, the Global Physical Activity Questionnaire (GPAQ) was completed. Spearman correlation coefficients and Bland-Altman analysis were used to assess construct validity.

**Results:**

Overall, 50 active adults (11 women, mean age = 25.1 ± 2.5) participated. Relative agreements between Total PA of PAQ24 and accelerometer were 0.37 ≤ ρ ≤ 0.72 for each day with satisfying agreement on five out of seven days. Weekly relative agreement for Total PA was moderate (*ρ* = 0.44). Relative agreements between PAQ24 and GPAQ were *ρ* = 0.43 for Total PA. Daily and weekly absolute agreements were poor indicated by wide limits of agreement.

**Conclusions:**

In contrast to weekly Total PA, the majority of daily results of the PAQ24 showed satisfying construct validity. A short recall period may improve the measurement quality of PA questionnaires, but measurement errors and the costs of multiple administrations must be considered in future studies.

## Background

Physical activity (PA) has been linked to a great number of physical and mental health benefits [1]. For example, there is strong evidence that moderate-to-vigorous physical activity (MVPA) reduces the risks of type 2 diabetes, coronary heart disease, depression and all-cause mortality [2, 3]. To achieve substantial health benefits, adults should perform at least 150 min of moderate-to-vigorous intensity aerobic activities per week as well as muscle-strengthening activities on two or more days per week [4]. This evidence is often based upon large cohort studies or randomized controlled trials using self-reported PA. However, the conclusions drawn from these studies depend on the quality of the assessment of exposures and outcomes.

For a long time, PA was exclusively assessed by self-report measures (e.g., questionnaires, diaries) due to the lack of alternatives. Today, research can rely on device-based measures such as accelerometers. Since accelerometry is not always feasible in large epidemiological studies and because questionnaires still provide important information about type (e.g., walking, cycling) and domains of PA (e.g., home, leisure time), questionnaires remain popular to gather valuable information at low cost [5]. In fact, a large part of the evidence forming the basis of current PA guidelines is based on questionnaire data [1]. Until now, many questionnaires have been developed since no gold standard for the measurement of PA exists [6].

Despite the existence of many different questionnaires, no conclusive recommendations can be provided for the best questionnaires to assess PA in various populations due to the inconsistent results regarding their measurement properties [7, 8]. The results for construct validity are often unsatisfying. Average correlations with accelerometer data of *r* = 0.22 for moderate and *r* = 0.32 for vigorous PA were reported [8], and for total PA the coefficients ranged from 0.04 to 0.47 [9]. This means that there is at best only 25% shared variance between these two methods [10]. Moreover, it seems that over the last decades the measurement quality of PA questionnaires did not considerably improve, for example, when comparing newly developed with already existing versions [11].

One shortcoming of a PA questionnaire is the reporting error associated with the recall period [12]. In a typical administration, a person is asked to recall and summarize all physical activities performed in a defined period (e.g., the past/usual week or past month). This means that a person should be able to correctly report frequency, duration and intensity of PA over the defined period. However, the person’s PA level is determined not only by the true amount of PA but also by the ability to recall all relevant activities. The accuracy of the recall may further be influenced by the type of activity. For example, the recall may be more difficult for sporadic, brief and low-intensity activities [13, 14].

However, evidence is accumulating that also light PA provides important health benefits such as reductions in mortality risks and improved cardiometabolic health, especially for inactive populations [15–17]. Moreover, current PA guidelines emphasized that also incidental, intermittent activities (e.g., less than 10 consecutive minutes) provide health benefits [1, 4]. Many existing questionnaires do not capture these brief activities [8]. A short recall period may be needed to capture all these relevant activities and, thus, help to improve the quality of PA measurement using questionnaires. The advantages of a short recall period have been highlighted previously. For example, Matthews et al. [14] acknowledged that it will reduce the cognitive demands of the participants because the recall would strongly rely on the recollection of behaviors using episodic memories. Hence, the authors recommended using multiple short-term recalls to obtain more accurate behavior-disease associations.

Although shorter recall periods (e.g., previous day) provide the potential to limit reporting errors, they were typically only applied in diaries or records such as within the “Activities Completed Over Time in 24 Hours” (ACT24) [18]. These formats tend to show better agreement with device-based measures of PA (e.g., 0.48 ≤ *r* ≤ 0.60 for ACT24) but at the expense of high burden for participants. Therefore, feasibility on a weekly basis, for example using multiple measurements, in large studies is limited. Until now, only two PA questionnaires used a recall period of one day and both showed good agreements with accelerometers (e.g., *r* = 0.71 for the Danish Physical Activity Questionnaire and *r* = 0.74 for the Daily Activity Questionnaire) [19, 20].

Since these two questionnaires are either too long or developed for a population of patients (i.e., after total hip arthroplasty), the measurement properties of a short daily PA questionnaire in a non-patient population are unknown. A short version could also help to increase feasibility when using multiple measurements. Therefore, the aim of this study was to assess the construct validity of a short self-administered daily PA questionnaire (Physical Activity Questionnaire for 24 h [PAQ24]) within a sample of the healthy population, namely young active adults. For the design of the questionnaire, we modified the International Physical Activity Questionnaire - Short Form (IPAQ-SF) and reduced the recall period from one week to one day.

Regarding PA, we hypothesized that the use of a daily recall period would: i) result in satisfying relative agreement between PAQ24 and other measures of the construct (established PA questionnaire, accelerometer). As recommended [8], we assumed correlations ≥0.50 between questionnaire and accelerometer and ≥ 0.70 between PA questionnaires as evidence for satisfying relative agreement; ii) result in satisfying absolute agreement between PAQ24 and these instruments. No thresholds for absolute agreement could be defined since there is no gold standard for the measurement of PA [6].

## Methods

### Study design and setting

In this cross-sectional study, recruitment of participants started on March 3, 2018 and was conducted at the Institute of Sport Science of the University of Graz, Austria. Participants of three different university courses were asked to participate in the study. Participants received no compensation but were able to request their individual results. Data collection was between May 4 and June 14, 2018. The initial assessments included anthropometric (measured weight and height, used to calculated body-mass-index [BMI, kg/m^2^]), sociodemographic data and individual aerobic capacity using the Chester Step Test [21]. This valid submaximal test was used to estimate maximum oxygen consumption (VO_2max_ [ml/kg/min]) and to describe the fitness level of the sample [22]. Participants completed the paper-pencil version of the PAQ24 at the end of each day for seven consecutive days and wore an accelerometer for the same period. In the second week, participants completed the Global Physical Activity Questionnaire (GPAQ) and a questionnaire obtaining participants’ experiences with the PAQ24 (i.e., What were your experiences with the questionnaire? Were there any difficulties when completing the questionnaire? What would you like to change, remove or add to the questionnaire?).

### Participants

Participants were students at the Institute of Sport Science and: (i) were registered for the programs sport science and/or physical education; (ii) had no self-reported acute physical injury; (iii) were fluent in the German language; and (iv) aged ≥18 years.

### Measurements

#### PAQ24

The IPAQ-SF – a validated questionnaire to assess PA in adults with reference to activities of the past or usual week [23] – was used to design our daily PA questionnaire (PAQ24). The PAQ24 aims to capture the total volume of PA (i.e., MVPA including walking) during the waking hours of a single day. Participants were instructed to complete the questionnaire before going to bed on seven consecutive days. As a reminder, a short message service (SMS) was provided every evening around 9 PM. The original German version of the PAQ24 (with a non-validated English translation) is included in the Additional file 1. The English version was translated by the authors for illustration purposes. Due to the lack of a correct translation process we do not recommend using this version.

We made three important changes compared to the IPAQ-SF. First, we changed the recall period to a 24 h day. Secondly, we included separate questions about resistance training, swimming, cycling for transport and cycling for exercise. The inclusion of resistance training should increase face validity since this is an essential part of current PA guidelines [1, 4]. Questions regarding swimming and cycling were included because they represent common activities and it is doubtful whether they can be appropriately detected by accelerometers [24]. Thirdly, instead of recalling only activities performed in bouts of at least 10 min, the PAQ24 asks participants to recall activities of any duration (i.e., all daily minutes). The questionnaire was pilot tested with 10 volunteers. Results were discussed and changes were made when required.

The questionnaire refers to all domains of PA (work, transport, recreation, sports, household/gardening) and obtains information regarding duration (minutes per day [min/day]), frequency (via the daily assessment) and intensity of PA with seven questions: walking, cycling (separate for transport and exercise), moderate activities and vigorous activities (separate for resistance training, swimming and any other vigorous activities). Two additional questions were included to assess both sedentary time (ST) and physical health status (illness, injury, no symptoms). In addition to the summarized score of all seven PA questions (Total PA), we also addressed the agreements of vigorous PA (VPA) and ST. VPA was calculated by summing up cycling for exercise and the three questions about vigorous activities. Cycling for exercise (e.g., racing) was considered as vigorous intensity according to the Compendium of Physical Activities [25] and other empirical investigations showing MET values ≥6 when cycling in higher speeds, even in trained individuals [26]. Although this study targeted the construct PA, results for ST were presented to allow comparisons with other studies. For additional analyses, the scores Total PA excluding walking, Total PA excluding cycling and Total PA excluding swimming were calculated. This was done due to the limited ability of the accelerometer to detect these activities and potential difficulties in the recall of all walking minutes [14, 24]. The scoring protocol of the PAQ24 can be found in the Additional file 1. All scores were expressed as min/day. Control of plausibility was based on the International Physical Activity Questionnaire (IPAQ) guidelines [27].

#### GPAQ

The GPAQ was developed by the World Health Organization (WHO) and is a 16-item validated instrument to assess PA in a usual week within three domains (recreation, work, transport) as well as ST [28]. We used the German version of the questionnaire and performed data cleaning and analysis according to the provided analysis guide [29]. The following scores were calculated: Total PA, VPA (derived from all three domains) and ST. All scores were expressed as min/day.

#### Accelerometer

Accelerometers are motion sensors which are able to measure acceleration, i.e. the change in velocity of an object over time [30]. The devices are worn on certain parts of the human body (e.g., hip, wrist, ankle) and measure the acceleration of the respective body segments during movement. Although, accelerometry has limitations when measuring activities that are highly static or insufficient captured due to body placement (e.g., wrist-worn while cycling), its recorded data (units of acceleration due to gravity) can be used to derive the frequency, duration and intensity of PA [31, 32].

We used GENEActiv® (Activinsights Ltd., Kimbolton, UK) accelerometers for the device-based measurement of participants’ PA. This triaxial device is water resistant and has a dynamic range of ±8 g. Participants were instructed to wear the accelerometer, in shape and dimension of a conventional watch, on their non-dominant wrist for 24 h per day for seven consecutive days. In addition, participants recorded any non-wear periods in a diary. Acceleration was recorded at 100 Hz and raw data was extracted using GENEActiv PC software version 3.2. Signal processing was performed in R (version 3.5.1; http://cran.r-project.org) using package GGIR (version 1.6–7).

Verification of sensor calibration error was performed and files were considered adequate for analyses if post-calibration error was less than 0.02 g [33, 34]. Non-wear was classified for a moving window of 60 min (with 15 min increments) if the standard deviation was less than 13 milligravity units (mg) or the range of values was less than 50 mg for at least two out of three axes [33, 35]. This window assures that short periods of sleep or inactivity were not misclassified as non-wear [35]. In presence of misclassification, the time stamp of the inactivity periods in addition to the information provided in the diary was used to determine non-wear periods more precisely. The algorithm to detect periods of inactivity was described previously [36]. Each accelerometer file was visually checked by two researchers. The vector magnitude (expressed in mg) using the Euclidean norm minus 1 g (ENMO: $\sqrt{a_x^2+{a}_y^2+{a}_z^2} - 1\ g$) was calculated based on 5 s epochs [35]. Any negative values were rounded up to zero. Because most participants started wearing the device in the morning of the first day, we considered 5 AM as the start of the measurement. For each day, sleep duration was estimated using a heuristic algorithm looking at the Distribution of Change in Z-Angle (HDCZA) [37]. Finally, total waking time was calculated as 24 h minus sleep duration. A day was considered as valid when waking non-wear time was less than 20% of the total waking time.

Thresholds for moderate and vigorous PA were considered as 100 mg and 400 mg, respectively [38]. Inactivity was defined as any time < 50 mg excluding sleep and non-wear [38, 39]. Unbouted time spent in different intensity categories was calculated (i.e., based on all 5 s epochs; expressed in min/day). Variables included in the analyses were Inactivity (< 50 mg), Total PA (≥ 50 mg), MVPA (≥ 100 mg), VPA (≥ 400 mg). Relative frequency of time spent in each intensity category based on the time the accelerometer was worn was multiplied by the total waking wear time to obtain non-wear adjusted minutes of PA and Inactivity.

### Sample size

Minimum coefficients for sufficient construct validity have been suggested [7, 8]. To detect the minimum correlation of 0.4 between scores from the questionnaire and accelerometer with a power of at least 80% (α = 0.05, two-tailed), a sample size of *n* ≥ 46 was required as calculated using G*Power version 3 [40]. This sample size will also allow sufficient precision in the estimation of the effect (i.e., 95% confidence interval [CI] with a maximum width of ±0.25) [41].

### Statistical analysis

Descriptive statistics are presented using median and interquartile range (IQR) for PA variables and mean and standard deviation for all other continuous variables (unless otherwise stated). The extent of agreement between variables of PAQ24 and accelerometer was assessed for each day (i.e., total minutes of each day) and the overall week (i.e., min/day as an average across the full measurement period). Daily and weekly scores of PAQ24 (Total PA, VPA, ST) were compared to related variables of GPAQ (Total PA, VPA, ST) and accelerometer (Total PA, MVPA, VPA, Inactivity). Concerning all daily comparisons between PAQ24 and accelerometer, only days with valid data in both instruments were included.

Spearman correlation coefficients (ρ) were used to determine the relative agreement. Weighted Spearman correlation coefficients were applied when there were missing days in either the accelerometer or PAQ24 assessment and weekly scores of the two instruments were addressed. A weighted analysis is recommended in presence of different numbers of repeated observations [42]. Bland-Altman analysis including mean difference and 95% limits of agreement (LOA) was used to evaluate absolute agreement [43]. Spearman correlations coefficients between weekly absolute differences and the average (ρ_diff_) were reported for Total PA, VPA and ST. Finally, mean differences were expressed as percentage (%), showing either average over- or under-reporting.

Sensitivity analyses were performed using the additional scores of the PAQ24 to assess whether agreements vary depending on the questionnaire score and accelerometer intensity category. Therefore, the agreement with the accelerometer was assessed for Total PA excluding walking, Total PA excluding cycling and Total PA excluding swimming. These scores as well as Total PA of the PAQ24 were also compared to both Total PA (≥ 50 mg) and MVPA (≥ 100 mg) from the accelerometer. Mann-Whitney U tests were used to compare included and non-included participants (e.g., due to missing accelerometer data) regarding sociodemographic characteristics and self-reported PA within the PAQ24. Statistical analyses were performed using SPSS Statistics version 25 (IBM Corp, Armonk, NY, USA) and GraphPad Prism version 7 (GraphPad Software, La Jolla, CA, USA).

## Results

### Participants and descriptive data

Of 70 students invited, 50 agreed to participate. All participants were found to be eligible and completed all parts of the study. In total, 39 (78%) men and 11 (22%) women participated (mean age = 25.1 ± 2.5, mean BMI = 23.7 ± 2.0). Mean aerobic capacity, represented by participants’ VO_2max_, was 50.6 ± 7.1 ml/kg/min (female: 44.9 ± 5.7, male: 52.3 ± 6.8).

All participants had valid data for all seven days of the PAQ24 measurement period, except for one participant who did not report ST on Monday. Among 350 days of accelerometry, six days were excluded due to technical problems and 24 days due to not meeting the criterion for a valid day. Three of the 50 participants had less than five valid days, whereas four participants had five valid days and 12 participants had six valid days. As a result, a total of 320 days (319 for ST) were included in all analyses comparing PAQ24 and accelerometer. Two participants reported implausible high values in the GPAQ and, thus, 48 (47 for ST) participants were included in all analyses comparing PAQ24 and GPAQ.

Table 1 shows minutes of daily and weekly PA and ST scores of PAQ24 and corresponding measures from the accelerometer. Minutes of all additional scores of the PAQ24 (i.e., Total PA excluding walking [MVPA], Total PA excluding cycling, Total PA excluding swimming) are provided in the Additional file 2. Participants reported a median duration of 1519 min (IQR: 1015–2203) of Total PA over the full measurement period with an average of 234 ± 97 min/day. Participants with missing accelerometer data on Saturday (*n* = 9) or Sunday *(n* = 7) were comparable to included participants regarding age, BMI and Total PA of PAQ24 (*p* > 0.05). Median accelerometer waking non-wear was 12 min/day (IQR: 0–35).

**Table 1:** Daily and weekly minutes of PA, ST and Inactivity measures from PAQ24 and accelerometer

	PAQ24			ACC			
	Total PA	VPA	ST	Total PA	MVPA	VPA	Inactivity
Monday	213 (140–289)	45 (0–91)	380 (240–455)^a^	296 (182–361)	154 (98–213)	8 (2–28)	622 (512–654)^a^
Tuesday	200 (148–278)	60 (0–85)	360 (240–510)	271 (214–379)	148 (99–192)	9 (3–23)	685 (599–736)
Wednesday	205 (130–340)	0 (0–60)	330 (240–510)	290 (230–366)	159 (115–208)	13 (3–27)	662 (605–727)
Thursday	168 (106–360)	30 (0–75)	360 (240–472)	292 (212–387)	155 (101–213)	10 (2–26)	679 (579–767)
Friday	220 (161–351)	60 (0–103)	360 (203–555)	295 (227–406)	146 (120–230)	8 (4–16)	667 (558–758)
Saturday	230 (160–330)	40 (0–90)	270 (180–390)	286 (236–375)	140 (116–184)	10 (4–21)	662 (586–755)
Sunday	155 (95–245)	0 (0–70)	330 (248–460)	260 (198–333)	127 (87–185)	8 (2–21)	693 (588–767)
Average/day	234 ± 97	52 ± 36	366 ± 137	298 ± 67	159 ± 45	15 ± 9	656 ± 81

Notes: All data presented in minutes using median (IQR) or mean ± SD based on either 46 (Monday), 49 (Tuesday), 47 (Wednesday), 48 (Thursday), 46 (Friday), 43 (Saturday), 41 (Sunday) or 50 (average per day) participants. *ACC* accelerometer, *IQR* interquartile range, *MVPA* moderate-to-vigorous physical activity, *PA* physical activity, *PAQ24* Physical Activity Questionnaire for 24 h, *SD* standard deviation, *ST* sedentary time, *VPA* vigorous physical activity. ^a^ based on 45 participants due to four invalid days in the accelerometer assessment and another missing value in the PAQ24

### Experiences with the PAQ24

Some participants reported that the recall of ST and walking time was difficult. Another common answer was the difficulty to report the total amount of active and inactive minutes and to differentiate between different intensities of PA. Participants’ recommendations to improve the questionnaire were to provide better explanations for moderate and vigorous PA, more examples and the opportunity to complete the questionnaire more than once per day.

### Comparison between questionnaires

In the GPAQ, participants reported a median duration of 157 min/day (IQR: 102–240) of Total PA, 51 min/day (IQR: 30–86) of VPA and 360 min/day (IQR: 270–480) of ST. Results for relative and absolute agreement between the questionnaires are reported in Table 2. A correlation of *ρ* = 0.43 was observed for Total PA. Compared to the GPAQ, the PAQ24 under-reported VPA on average by 20% and ST by 0.3% whereas over-reported Total PA by 36%.

**Table 2:** Relative and absolute agreement of Total PA, VPA and ST between PAQ24 and GPAQ

	Relative Agreement		Absolute Agreement		
	ρ (95% CI)	*p*	M_diff_ ± 95% LOA	ρ_diff_ (95% CI)	*p*
Total PA	0.43 (0.16–0.64)	0.002	−63 ± 210	−0.10 (−0.38–0.19)	0.48
VPA	0.51 (0.26–0.70)	< 0.001	13 ± 91	0.23 (−0.06–0.48)	0.12
ST	0.74 (0.58–0.85)	< 0.001	1 ± 165	0.07 (−0.23–0.35)	0.66

Notes: Results were based on either 48 (Total PA, VPA) or 47 (ST) participants. Relative agreement was assessed by Spearman correlations coefficients (ρ) and absolute agreement by Bland-Altman analyses including mean difference and 95% limits of agreement (reported as minutes per day) and corresponding Spearman correlation coefficients between absolute differences and the average of the two questionnaires (ρ_diff_). *CI* confidence interval, *GPAQ* Global Physical Activity Questionnaire, *LOA* limits of agreement, *PA* physical activity, *PAQ24* Physical Activity Questionnaire for 24 h, *ST* sedentary time, *VPA* vigorous physical activity

### Comparison with accelerometer

**Fig. 1:**
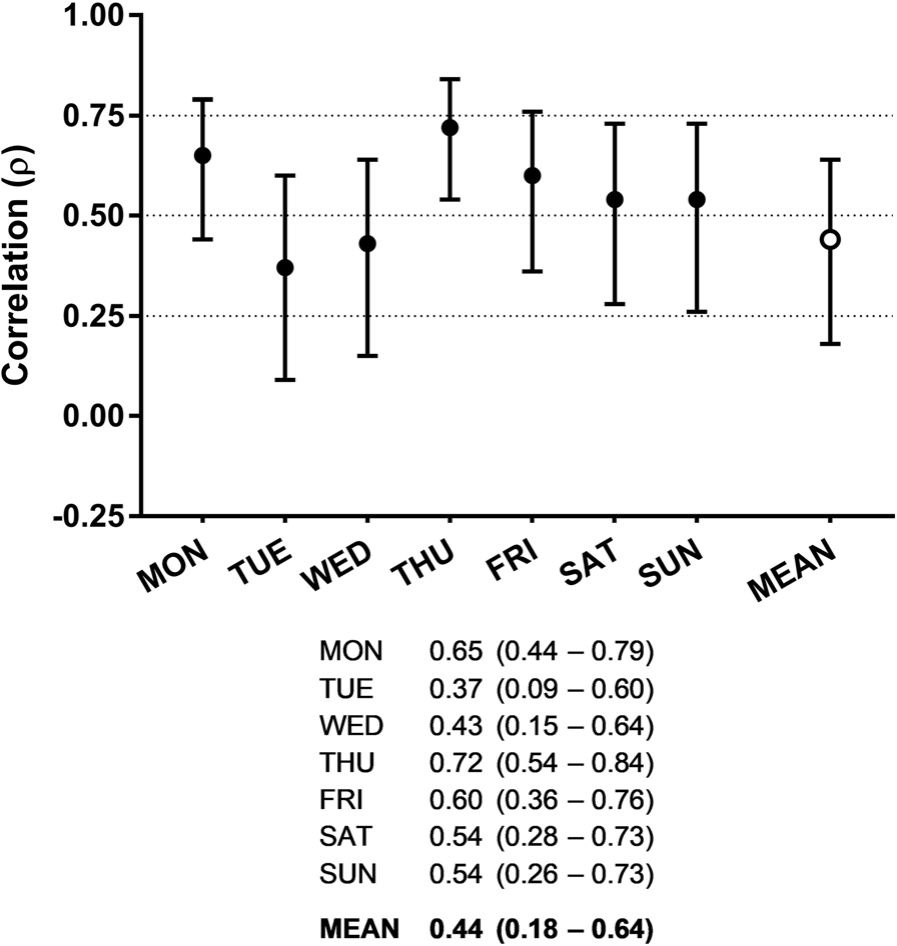
Summary of daily and weekly relative agreement of Total PA between PAQ24 and accelerometer. Results are presented as Spearman correlation coefficients with 95% confidence intervals. *PA* physical activity, *PAQ24* Physical Activity Questionnaire for 24 h

**Fig. 2:**
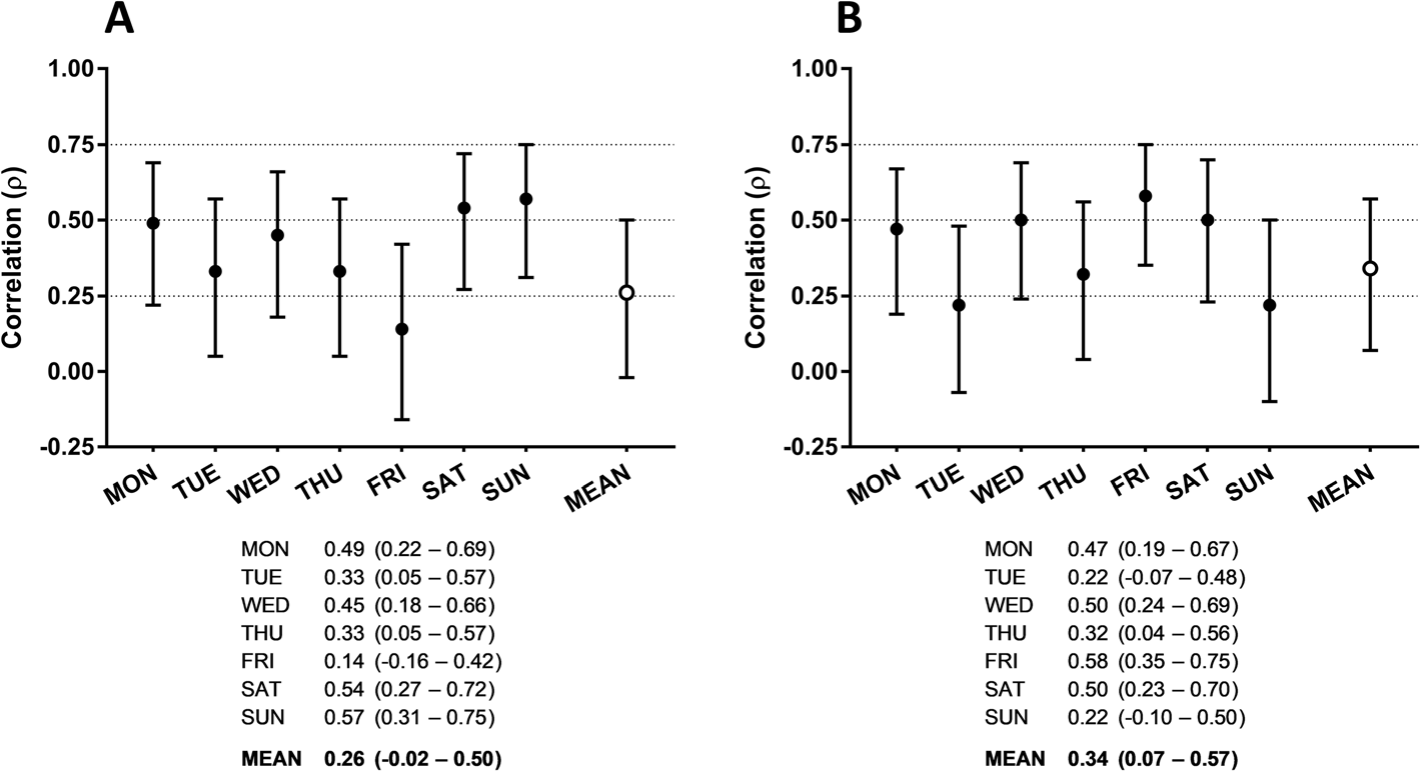
Summary of daily and weekly relative agreement of VPA (**a**) and ST (**b**) between PAQ24 and accelerometer. Results are presented as Spearman correlation coefficients with 95% confidence intervals. *PAQ24* Physical Activity Questionnaire for 24 h, *ST* sedentary time, *VPA* vigorous physical activity

Daily and weekly relative agreements of Total PA, VPA and ST of PAQ24 with corresponding measures from the accelerometer (Total PA, VPA, Inactivity) are shown in Figs. 1 and 2. Correlation coefficients for daily Total PA ranged from 0.37 to 0.72 with correlations of ≥0.50 on five out of seven days. For the overall week, the highest correlation was observed for Total PA (*ρ* = 0.44, *p* = 0.002).

**Fig. 3:**
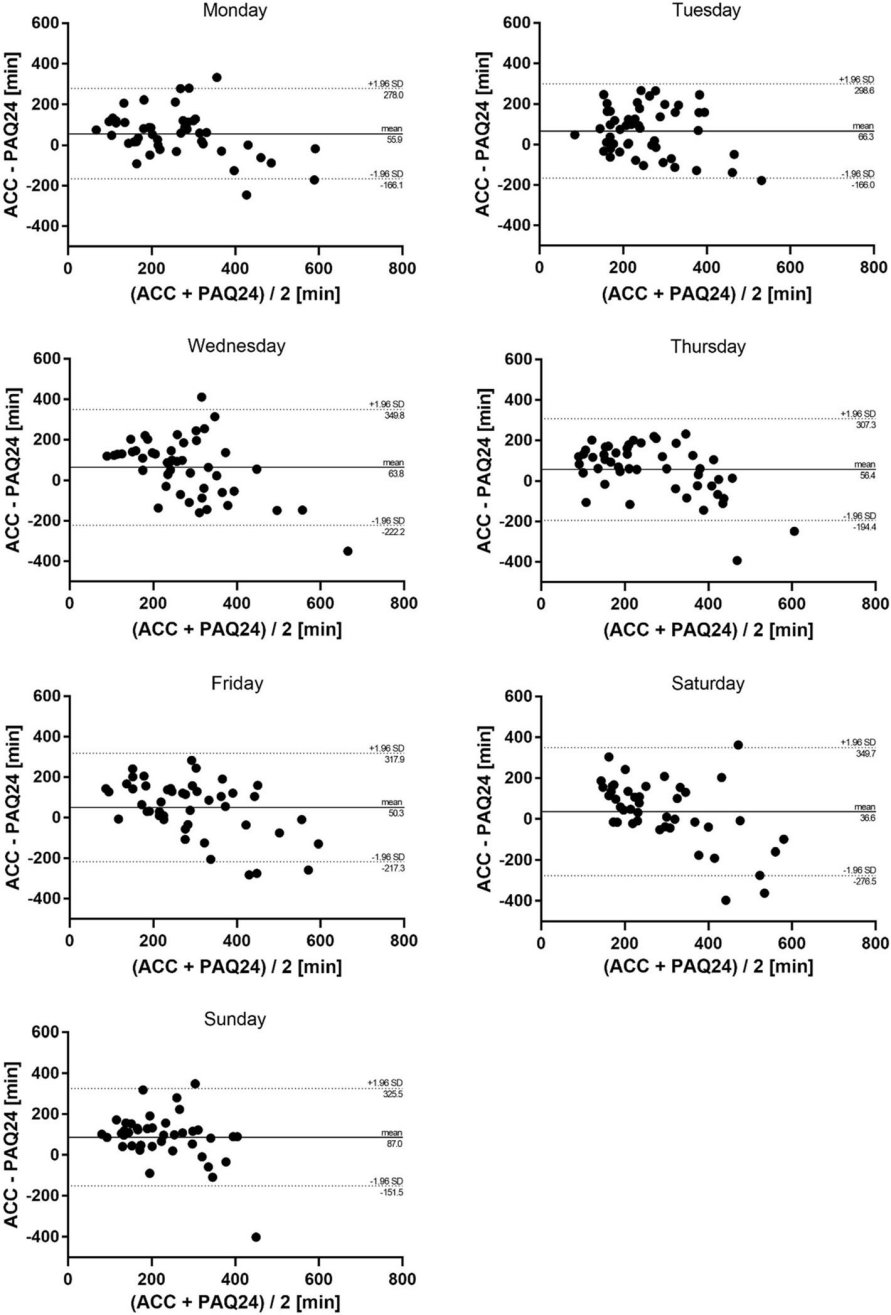
Absolute agreement of daily Total PA between PAQ24 and accelerometer. Bland-Altman plots for each day showing difference versus average of the values measured by the two methods with 95% limits of agreement. *ACC* accelerometer, *PA* physical activity, *PAQ24* Physical Activity Questionnaire for 24 h

Figure 3 shows mean differences and 95% LOA of Total PA for each day. The smallest 95% LOA were observed on Monday (56 ± 222 min). A positive mean difference (i.e., higher accelerometer values) was evident for all days ranging from 37 to 87 min. These mean differences showed that daily Total PA was consistently under-reported by the PAQ24 (range: 12 to 32%).

**Fig. 4:**
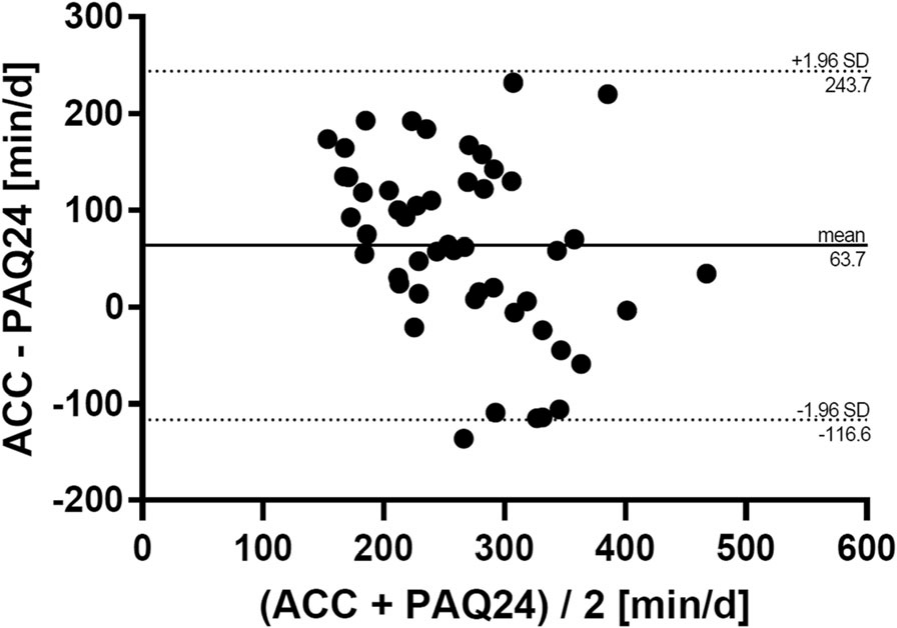
Absolute agreement of weekly Total PA between PAQ24 and accelerometer. Bland-Altman plots for each day showing difference versus average of the values measured by the two methods with 95% limits of agreement. *ACC* accelerometer, *PA* physical activity, *PAQ24* Physical Activity Questionnaire for 24 h

Similar 95% LOA were observed for weekly Total PA (Fig. 4) including a correlation of ρ_diff_ = − 0.42 (95% CI: − 0.63 – − 0.16; *p* = 0.002) between the absolute differences and the average. Compared to the accelerometer, the PAQ24 under-reported weekly Total PA by 21% on average per day. Results of absolute agreement for daily and weekly VPA and ST are shown in the Additional files 3 and 4. Absolute agreement was − 36 ± 70 min/day (ρ_diff_ = − 0.85, 95% CI: − 0.91 – − 0.74; *p* <  0.001) for weekly VPA and 290 ± 256 min/day (ρ_diff_ = − 0.45, 95% CI: − 0.65 – − 0.20; *p* = 0.001) for weekly ST. This means that, compared to the accelerometer, the PAQ24 over-reported weekly VPA by 338% and under-reported weekly ST by 44% on average per day.

Results of all additional analyses are reported in the Additional files 5 and 6 (relative agreement) and Additional file 7 (absolute agreement). These files also show the results for the agreement between PAQ24 and aerobic capacity (e.g., *ρ* = − 0.07 for Total PA). Compared to weekly Total PA, similar correlations were observed for weekly Total PA excluding walking (*ρ* = 0.38), weekly Total PA excluding cycling (*ρ* = 0.41) and weekly Total PA excluding swimming (*ρ* = 0.41). Comparing weekly Total PA of the PAQ24 with MVPA instead of Total PA from the accelerometer resulted in a lower relative agreement (*ρ* = 0.29). This was similar, although usually less pronounced, for the additional scores of the PAQ24. When comparing weekly Total PA of PAQ24 with MVPA, instead of Total PA, from the accelerometer, absolute agreements changed. The width of the 95% LOA was similar but changes in the mean difference were observed (− 75 ± 183 min/day; ρ_diff_ = − 0.65, 95% CI: − 0.79 – − 0.46; *p* <  0.001). Similar results for absolute and relative agreements were obtained for each day.

## Discussion

The purpose of this study was to assess the construct validity of a short self-administered daily PA questionnaire (PAQ24) in young active adults. We expected that the short recall period will result in satisfying construct validity. However, the results of the study revealed inconclusive evidence for the construct validity of the PAQ24. Compared to accelerometry, the PAQ24 showed satisfying relative agreements (i.e., ρ ≥ 0.5) on five out of seven days when assessing Total PA. The relative agreements for the overall week (i.e., averages per day) were unsatisfying for all scores, including Total PA. Similar moderate, but not satisfying (ρ <  0.70), agreements were observed when comparing scores of PAQ24 and GPAQ. Furthermore, absolute agreements for both daily and weekly scores were poor because of wide LOA. Additional analyses using different scores of the PAQ24 or accelerometer intensity cut points resulted in similar or lower agreements.

PA reported in the PAQ24 varied from day to day with the highest minutes on Saturday and the lowest on Sunday. This variation may be influenced by daily differences in the amount of leisure time, participation in sport events or convenience of scheduling [44]. For daily PA assessments, it is important to consider this variation in PA [45]. In general, the use of multiple short-term measurements should increase the ability of the instrument to distinguish between true variation in PA and other sources of error [46]. However, depending on the data collection method, study population and choice of PA score under investigation, the number of measurements needed to capture this true variation in PA may vary. For example, three to five days of accelerometer monitoring might be needed to obtain accurate levels of PA in adults [45, 47] but more days are required for the assessment of Inactivity [48], when using self-report methods [46], or in specific populations such as children [49]. The impact of the variance in PA behaviors on reliability and the minimum number of measurements when using daily PA questionnaires such as the PAQ24 must be evaluated in future studies. This would also help in drawing conclusions about the feasibility of such a questionnaire.

Overall, the daily results for Total PA were comparable to more sophisticated 24 h diaries and recalls [8, 11, 18, 50]. The relative agreement of weekly Total PA between PAQ24 and accelerometer did not meet our criterion for satisfying validity but was in the upper range of results when using previous questionnaires [8, 11, 51]. For example, a systematic review of 23 studies on the construct validity of the IPAQ-SF reported correlations ranging from 0.09 to 0.39 for total PA when compared to device-based measures of PA [51]. Absolute agreement of weekly PA scores of the PAQ24 was rather poor. We observed smaller LOA for weekly compared to daily scores due to reduced random error when using averages of multiple measurements. Moreover, increases in Total PA and VPA were associated with changes in the observed difference between PAQ24 and accelerometer (e.g., shifted from under- to over-reporting with increasing PA scores).

The lack of agreement between PAQ24 and accelerometer may be attributable to differences in individual characteristics and the measurement quality of both methods. For example, it has been shown that brief, unstructured or low-intensity activities are difficult to recall [13, 14] and that the level of agreement varies depending on factors such as age, weight-status or accelerometer data processing [52]. Our participants perceived difficulties with classifying the intensity of activities and with reporting the total volume of walking time. On the other side, acceleration of activities such as cycling or resistance training may not be able to be accurately captured by device-based measurement [24]. However, no improvements in the agreement were observed after excluding these activities. We also observed daily variation in the agreements, which could be influenced by both random and systematic error. For example, activities which are poorly detected by the accelerometer may have been performed on specific days. Likewise, some days may include more structured activities and events (e.g., exercise sessions, competitions) which are easier to recall [13].

Neither questionnaires nor accelerometers are perfect tools to measure PA. This lack of real gold standard was correctly acknowledged by several researchers [6, 53]. In addition to the disadvantage of reporting errors, a further limitation is, that questionnaires are always developed for a specific population (e.g., elderly, adults, youth, pregnancy) and the identification of most qualified ones is difficult [7, 8, 54, 55]. Moreover, the interpretation of questions in the questionnaire (e.g. intensity description) is influenced by characteristics of the participant such as perceived confidence [56] and origin (e.g., different countries and cultures need cross-cultural adaptations) [57]. These individual characteristics can limit the measurement quality and may result in an under- or overestimation of self-reported PA. On the other side, PA data derived from accelerometry is influenced by several decisions of the researcher. Depending on brand [58], body placement [59, 60] and sampling frequency [47], the data, used for subsequent analyzing, is already affected by researchers’ pre-choices. Also, several other decisions (e.g., intensity cut points, epoch length, filters, algorithms to detect non-wear, requirements for a valid day/week) have been shown to influence the PA estimates from the accelerometer [47, 61]. The current lack of consensus on best practices to handle accelerometer data hampers the quality of the assessment of measurement properties of PA questionnaires (since accelerometers are often considered as “reference” measure).

The agreements for the overall week between PAQ24 and accelerometer were lower than what we would assume based on the pattern in the daily results. This may be due to a stronger influence of different systematic (e.g., additive and multiplicative) errors [62]. For example, consistent under- or over-reporting of PA can influence the estimation of mean, dispersion or participants’ ranking order when considering averages per day. Such a reporting bias may only exist for some but not all participants (differential recall bias) and can either increase or decrease the level of agreement [63]. Furthermore, our results showed that the true level of PA was also related to the level of agreement, namely by changes in over- and under-reporting with different PA levels (see results from Bland-Altman analysis). These influences on the repeatability of the tools could have reduced the weekly compared to daily relative agreements, even if one instrument would be free of error [64].

The results also demonstrated poor absolute agreements and only moderate correlations between PAQ24 and GPAQ. Neither Total PA nor VPA did meet our criterion for satisfying construct validity. However, similar correlations were reported in previous studies when comparing forms of the IPAQ with the GPAQ [65, 66]. This lower agreement can be influenced by differences in the questionnaire format. The PAQ24 includes separate questions for cycling, walking, swimming and resistance training whereas the GPAQ combines these activities into fewer questions and obtains information using different domains of PA [28]. The use of different recall periods (e.g.,” typical week” in the GPAQ) could also have reduced the level of agreement [67].

ST of the PAQ24 was strongly related to ST of the GPAQ but less to Inactivity from the accelerometer. Also, Bland-Altman analyses indicated poor absolute agreements for daily and weekly ST which seems to be in line with previous results showing usually an under-reporting of ST compared to the accelerometer [52]. This poor agreement may be partly explained by difficulties in reporting ST, as mentioned by some participants, and the lower accuracy of wrist-worn accelerometers (without further use of inclinometers) to differentiate between non-movement positions such as lying, sitting or standing [68]. However, the results of weekly ST are comparable to previous questionnaires [11]. Finally, PA was not associated with aerobic capacity (see Additional file 5) which may be due to the non-overlapping parts of the two concepts [69], the variability in PA [46] and the homogeneity of the sample regarding their usually high fitness levels.

The results of the present study must be interpreted with respect to our specific sample, since participants involved were highly active and trained students. The participants were affine to sports and exercise, and therefore, should be able to better estimate the intensity of PA compared to a sample with a different background. Many participants were members of a sports club with settled weekdays of training and were registered for obligatory university exercise courses. Taking this into account, it might have been easier for them to recall PA, compared to the general population. This strongly limits the generalizability of our results. Future studies are therefore needed to evaluate the PAQ24 and other promising daily PA questionnaires in representative samples of the general population.

Finally, we tried to improve the measurement properties of PA questionnaires by using a short recall period. Although we modified an existing questionnaire for our purposes, we do not recommend using the PAQ24 to measure PA in other studies. Already in 2000, Sallis and Saelens [5] recognized the existence of too many different questionnaires and recommended to use only the most qualified ones for future research. Therefore, we strongly recommend using an existing questionnaire whenever possible. The choice of the questionnaire should follow the purpose of the study and the evaluation of measurement properties (e.g., content validity, reliability, construct validity, responsiveness). Several reviews on measurement properties of PA questionnaires have been published [7, 8, 11, 53, 70] and may help in the selection of most qualified questionnaires. However, we invite researchers to use our questionnaire in future validation studies to further improve the measurement quality of PA questionnaires. For example, using smartphone applications for the daily assessment may increase feasibility. Future studies should also evaluate the measurement errors associated with multiple measurements as well as minimum required days of monitoring. Overall, we, together with others [14], argue that multiple short-term recalls are a promising approach to overcome important short-comings of traditional PA questionnaires.

### Strengths and limitations

First, the specific study population (young active adults) limits the generalizability of the findings. Secondly, although participants were instructed to complete the PAQ24 before going to bed, we did not assess whether they were still awake and active after they completed the questionnaire. Thirdly, results for absolute agreement showed a strong dependence on accelerometer intensity cut points which should be considered when interpreting the results. This seems reasonable when using lower or higher cut points and was rather affecting the mean difference than the magnitude and variation of differences (i.e., LOA). Finally, this study did not assess the effect of a short recall period using an experimental design comparing it with a recall period of a week. On the other side, the study has several strengths: i) the use of raw accelerometry to increase transparency and comparability between studies; ii) reporting the influence of different accelerometer intensity cut points on the results; iii) the use of guidelines regarding the validation of PA questionnaires (e.g., specifying a priori hypotheses) [7, 8, 71]; and iv) data collection was performed within a short period, which reduces the influences caused by changes in weather, seasons or types of activities.

## Conclusions

A short recall period may improve the measurement quality of PA questionnaires as this was seen in the daily results for Total PA (five out of seven days showed sufficient construct validity). In contrast, the weekly results for Total PA did not meet our criterion. Also, agreements with the GPAQ were unsatisfying and absolute agreements were poor. Since the results of the present study were based on a very specific sample, studies in representative samples of the general adult population are needed. Also, the feasibility of a short daily PA questionnaire in large studies as well as the influence of measurement errors should be evaluated in future studies. Altogether, the results of the PAQ24 are promising and researchers are invited to use our questionnaire in future studies exclusively for the purpose of improving the measurement quality of PA questionnaires (e.g., to use smartphone applications for the daily assessment).

## Supplementary information

**Additional file 1.** German version (original) and translated English version (not validated) of the PAQ24.

**Additional file 2.** Daily and weekly minutes of all additional scores of the PAQ24.

**Additional file 3.** Absolute agreement of daily and weekly VPA between PAQ24 and accelerometer. Bland-Altman plots for each day showing difference versus average of the values measured by the two methods with 95% limits of agreement.

**Additional file 4.** Absolute agreement of daily and weekly ST between PAQ24 and accelerometer. Bland-Altman plots for each day showing difference versus average of the values measured by the two methods with 95% limits of agreement.

**Additional file 5.** Relative agreement of Total PA, Total PA excl. Walking (MVPA), Total PA excl. Cycling and Total PA excl. Swimming between PAQ24 and different measures of the accelerometer.

**Additional file 6.** Relative agreement of VPA and ST between PAQ24 and accelerometer.

**Additional file 7.** Absolute agreement of Total PA, Total PA excl. Walking (MVPA), Total PA excl. Cycling and Total PA excl. Swimming between PAQ24 and different measures of the accelerometer.

## Data Availability

The datasets used and/or analyzed during the current study are available from the corresponding author on reasonable request.

## Abbreviations

ACC: Accelerometer
ACT24: Activities completed over time in 24 h
BMI: Body mass index
ENMO: Euclidean norm minus 1 g
GPAQ: Global Physical Activity Questionnaire
HDCZA: Heuristic algorithm looking at distribution of change in Z-Angle
IPAQ: International Physical Activity Questionnaire
IPAQ-SF: International Physical Activity Questionnaire – Short Form
LOA: Limits of agreement
MET: Metabolic equivalent
mg: Milligravity units
MVPA: Moderate-to-vigorous physical activity
PA: Physical activity
PAQ24: Physical Activity Questionnaire for 24 h
SMS: Short message service
ST: Sedentary time
VO2max: Maximum oxygen consumption
VPA: Vigorous physical activity
WHO: World Health Organization
