# Supplementary material for: Do we have to reduce the recall period? Validity of a daily physical activity questionnaire (PAQ24) in young active adults
Source: BMC Public Health. 2020 Jan 16;20:72. doi: 10.1186/s12889-020-8165-3 (PMC6966869; doi:10.1186/s12889-020-8165-3)
Supplement: Supplementary file 1 — Additional file 1. German version (original) and translated English version (not validated) of the PAQ24. [file 12889_2020_8165_MOESM1_ESM.docx]

**Additional file 1** German version (original) and translated English version (not validated) of the PAQ24, including the scoring protocol.

*Original German version:*

Wir sind daran interessiert herauszufinden, welche Arten von körperlichen Aktivitäten Menschen in ihrem täglichen Leben vollziehen. Die Befragung bezieht sich auf die Dauer, die Sie während des **heutigen Tages** in körperlicher Aktivität verbracht haben. Bitte beantworten Sie alle Fragen (auch wenn Sie sich selbst nicht als aktive Person ansehen). Bitte berücksichtigen Sie **alle** Aktivitäten wie beispielsweise im Rahmen Ihrer Arbeit, um von Ort zu Ort zu gelangen, zu Hause und im Garten sowie in Ihrer Freizeit für Erholung und Sport.

Denken Sie an all Ihre Aktivitäten des **heutigen Tages**. Diese können **leichte** Aktivitäten (z.B. Gehen) als auch a**nstrengende** und **moderate** Aktivitäten beinhalten. **Moderate** Aktivitäten bezeichnen Aktivitäten mit mittlerer körperlicher Anstrengung, bei denen Sie ein wenig stärker atmen als normal. **Anstrengende** Aktivitäten bezeichnen Aktivitäten, die starke körperliche Anstrengungen erfordern und bei denen Sie deutlich stärker atmen als normal.

1. Wie viel Minuten haben Sie **heute** mit **Gehen** verbracht? Diese beinhalten **alle** Gehstrecken zu Hause, im Garten, an der Universität, in der Arbeit, um von Ort zu Ort zu gelangen, in der Freizeit sowie alle anderen Gehstrecken.

Minuten  Keine entsprechenden Wege zu Fuß

1. Wie viel Minuten haben Sie **heute** mit **Radfahren** verbracht?

Minuten, um von Ort zu Ort zu gelangen (z.B. von zu Hause zur Arbeit)

Minuten zur körperlichen Betätigung (Mountainbike, Rennrad, Ausflug)

 Keine entsprechenden Wege mit dem Fahrrad

1. Wie viel Minuten haben Sie **heute** mit **Sitzen** verbracht? Diese beinhalten **alle** sitzenden Tätigkeiten zu Hause, im Garten, an der Universität, in der Arbeit, während von Ort zu Ort zu gelangen, in der Freizeit sowie alle anderen sitzenden Tätigkeiten.

Stunden und Minuten

Die nächsten zwei Fragen beziehen sich auf moderate (Frage 4) oder anstrengende (Frage 5) Aktivitäten. Bitte beachten Sie, dass die Minuten, die Sie heute mit Gehen und Radfahren verbracht haben bereits bei Frage 1 und 2 festgehalten wurden. **Bitte beantworten Sie daher die Fragen 4 und 5 ohne die Aktivitäten Gehen und Radfahren**.

1. Wie viele Minuten haben Sie heute mit **moderater** körperlicher Aktivität (ohne Gehen, Radfahren), bei denen Atmung und Puls leicht zunehmen, verbracht (z.B. Tragen leichter Lasten, Nordic-Walking, Arbeiten für den Haushalt oder Garten)?

Minuten  Keine moderate Aktivität

1. Wie viele Minuten haben Sie heute mit **anstrengender** körperlicher Aktivität (ohne Gehen, Radfahren), bei denen Atmung und Puls stark zunehmen, verbracht (z.B. Tragen schwerer Lasten, Bauarbeiten, Laufen, Fußball, Basketball, Ultimate Frisbee, Hockey)?

Minuten (ohne Krafttraining, ohne Schwimmen)

Minuten Schwimmen

________Minuten Krafttraining

 Keine anstrengende Aktivität

1. Wie ist Ihre derzeitige körperliche Verfassung?

 akute Erkrankung (z.B. Grippe, Infekt)

 akute Verletzung (z.B. Bänderzerrung, Knochenbruch)

 Keine Beschwerden

*Translated English version. We do not recommend using this non-validated version. This version was only translated by the authors for illustration purposes.*

We are interested in finding out what types of physical activity people perform in their daily lives. The survey refers to the amount of physical activity you have spent **during the day**. Please answer all questions (even if you do not consider yourself an active person). Please think about all the activities such as work, to get from place to place, at home and in the garden, and in your spare time for recreation and sport.

Think of all your **vigorous and moderate activities of the day.** Vigorous activities are activities that require a lot of physical effort and that give you a lot more breathing than normal. Moderate activities are activities with moderate physical effort, where you breathe a little more than normal.

1. During **today**, how many minutes did you spend with **walking**? This includes distances at home, garden, university, work, walking to travel from place to place or any other distances in your leisure time.

minutes  no distances walking

1. During **today**, how many minutes did you spend with **cycling**?

minutes, from place to place (e.g. home to work)

minutes as physical exercise (mountain bike, racing bicycle, tour)

 no distances cycling

1. During **today**, how many minutes did you spend with **sitting**? This include all sitting activities at home, in the garden, at the university, at work, while getting from place to place, during leisure, as well as all other sitting activities.

minutes  no sitting activities

The next two questions are related to **moderate** (question 4) or **vigorous** (question 5) **activities**. Please note that the minutes you spend today walking and cycling have already been recorded in question 1 and 2. **Please answer therefore Questions 4 and 5 without the activities walking and cycling.**

1. During **today**, how many minutes did you spend with **moderate** physical activities (without walking and cycling)? Moderate activities refer to activities that take moderate physical effort and make you breathe somewhat harder than normal (carrying light loads, Nordic walking, household and gardening.

minutes  no moderate activities

1. During **today**, how many minutes did you spend with **vigorous** physical activities (without walking and cycling)? Vigorous physical activities refer to activities that take hard physical effort and make you breathe much harder than normal (carrying heavy loads, digging, running, soccer, basketball, ultimate frisbee, hockey).

minutes (without weight training or swimming)

________minutes swimming

________minutes weight training

 no vigorous activities

1. What is your current **physical** **condition**?

 acute illness (flu, infect)

 acute injury (distortion of ligaments, bone fracture)

 no physical complaints

Scoring of the PAQ24

| **Name of variable** | **Questions** |
| --- | --- |
| Total PA | 1+2a+2b+4+5a+5b+5c |
| VPA | 2b+5a+5b+5c |
| Total PA excluding walking | 2a+2b+4+5a+5b+5c |
| Total PA excluding cycling | 1+4+5a+5b+5c |
| Total PA excluding swimming | 1+2a+2b+4+5a+5c |
| ST | 3 |

*PA* physical activity, *ST* sedentary time, *VPA* vigorous physical activity.
