# Supplementary material for: Do we have to reduce the recall period? Validity of a daily physical activity questionnaire (PAQ24) in young active adults
Source: BMC Public Health. 2020 Jan 16;20:72. doi: 10.1186/s12889-020-8165-3 (PMC6966869; doi:10.1186/s12889-020-8165-3)
Supplement: Supplementary file 2 — Additional file 2. Daily and weekly minutes of all additional scores of the PAQ24. [file 12889_2020_8165_MOESM2_ESM.docx]

**Additional file 2** Daily and weekly minutes of all additional scores of the PAQ24

|  | Total PA excluding walking | Total PA excluding cycling | Total PA excluding swimming |
| --- | --- | --- | --- |
| Monday | 100 (30 – 185) | 175 (88 – 249) | 213 (140 – 289) |
| Tuesday | 115 (73 – 155) | 155 (118 – 268) | 200 (148 – 278) |
| Wednesday | 90 (45 – 140) | 180 (90 – 320) | 195 (130 – 340) |
| Thursday | 105 (23 – 177) | 143 (80 – 360) | 163 (106 – 360) |
| Friday | 110 (34 – 214) | 218 (124 – 330) | 220 (162 – 351) |
| Saturday | 120 (40 – 180) | 215 (130 – 310) | 230 (160 – 330) |
| Sunday | 60 (30 – 133) | 135 (78 – 310) | 155 (95 – 245) |
| Average/day | 116 ± 97 | 213 ± 98 | 234 ± 96 |

Notes: All data presented in minutes using median (IQR) or mean ± SD based on either 46 (Monday), 49 (Tuesday), 47 (Wednesday), 48 (Thursday), 46 (Friday), 43 (Saturday), 41 (Sunday) or 50 (average per day) participants. *IQR* interquartile range, *PA* physical activity, *PAQ24* Physical Activity Questionnaire for 24 h, *SD* standard deviation.
