# Supplementary material for: Do we have to reduce the recall period? Validity of a daily physical activity questionnaire (PAQ24) in young active adults
Source: BMC Public Health. 2020 Jan 16;20:72. doi: 10.1186/s12889-020-8165-3 (PMC6966869; doi:10.1186/s12889-020-8165-3)
Supplement: Supplementary file 4 — Additional file 4. Absolute agreement of daily and weekly ST between PAQ24 and accelerometer. Bland-Altman plots for each day showing difference versus average of the values measured by the two methods with 95% limits of agreement. [file 12889_2020_8165_MOESM4_ESM.docx]

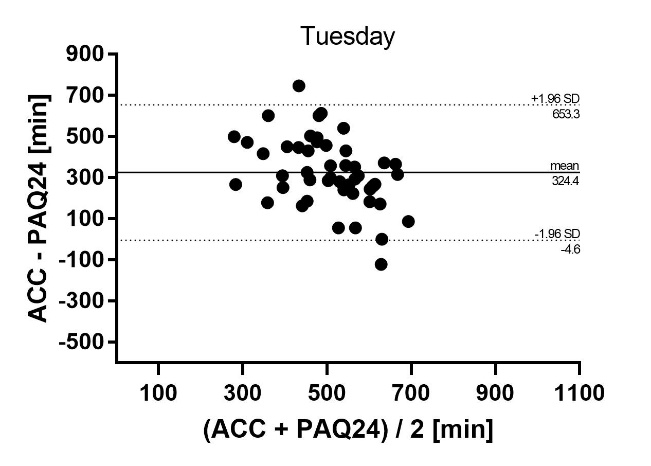

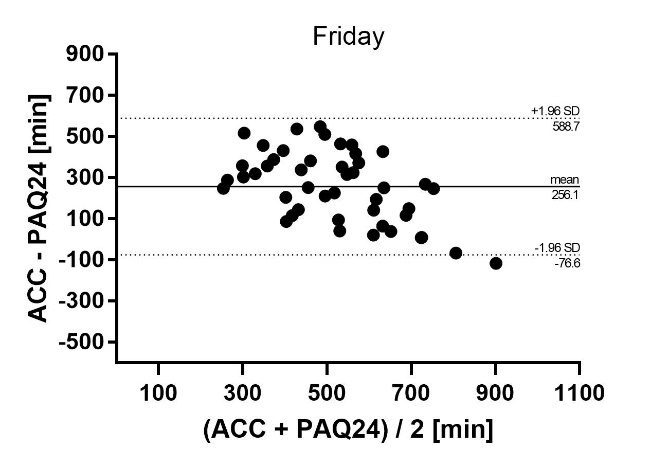

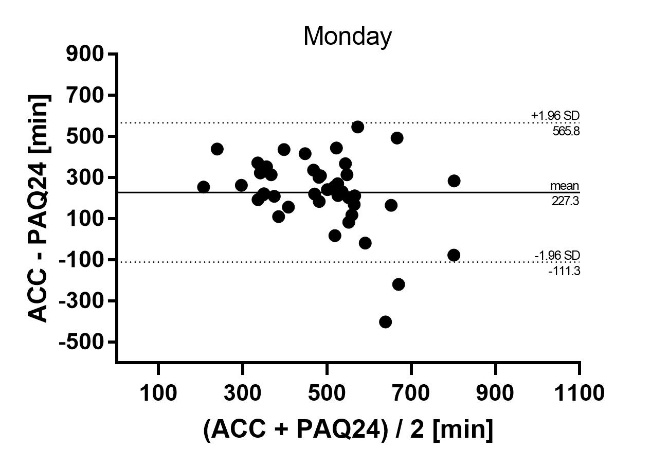

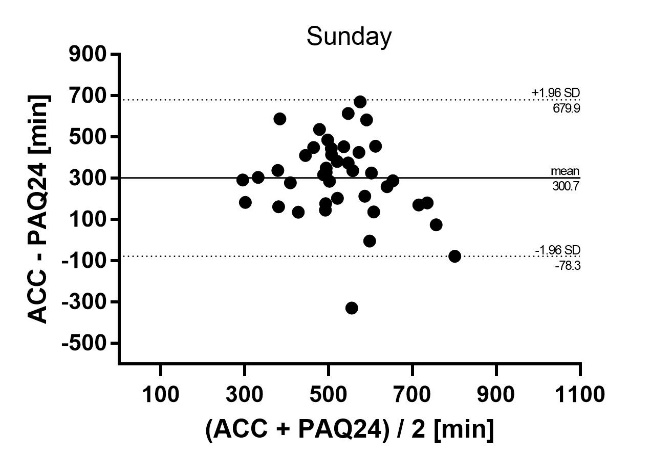

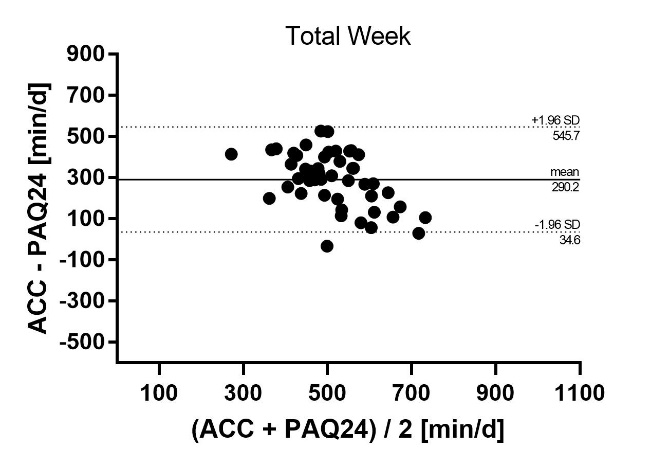

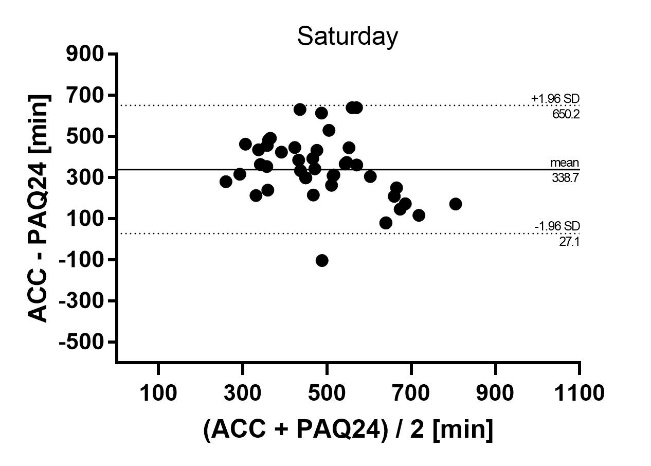

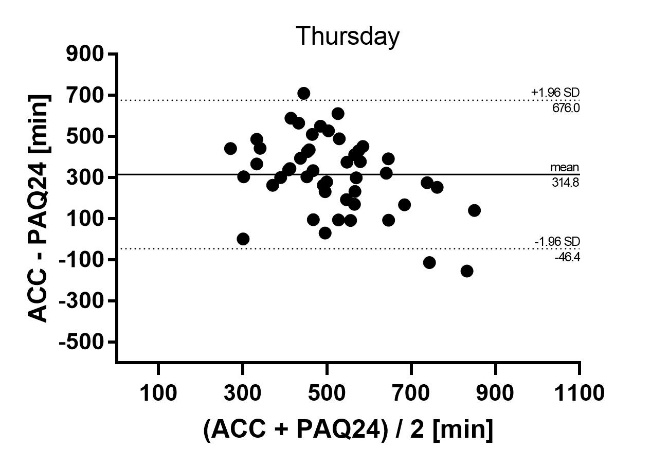

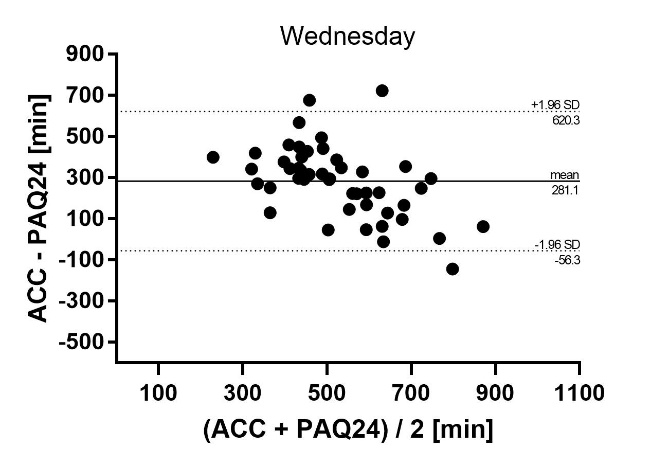


**Additional file 4** Absolute agreement of daily and weekly ST between PAQ24 and accelerometer. Bland-Altman plots for each day showing difference versus average of the values measured by the two methods with 95% limits of agreement. *ACC* accelerometer, *PAQ24* Physical Activity Questionnaire for 24 h, *ST* sedentary time
