# Supplementary material for: Do we have to reduce the recall period? Validity of a daily physical activity questionnaire (PAQ24) in young active adults
Source: BMC Public Health. 2020 Jan 16;20:72. doi: 10.1186/s12889-020-8165-3 (PMC6966869; doi:10.1186/s12889-020-8165-3)
Supplement: Supplementary file 5 — Additional file 5. Relative agreement of Total PA, Total PA excl. Walking (MVPA), Total PA excl. Cycling and Total PA excl. Swimming between PAQ24 and different measures of the accelerometer. [file 12889_2020_8165_MOESM5_ESM.docx]

**Additional file 5** Relative agreement of Total PA, Total PA excluding walking, Total PA excluding cycling and Total PA excluding swimming between PAQ24 and different measures of the accelerometer

|  |  | **Total PA** |  |  | **Total PA excluding walking** |  |  | **Total PA excluding cycling** |  |  | **Total PA excluding swimming** |  |
| --- | --- | --- | --- | --- | --- | --- | --- | --- | --- | --- | --- | --- |
|  | Comparison  measure | ρ (95% CI) | *p* |  | ρ (95% CI) | *p* |  | ρ (95% CI) | *p* |  | ρ (95% CI) | *p* |
| Monday | ACC Total PA | 0.65 (0.44 – 0.79) | < 0.001 |  | 0.61 (0.38 – 0.77) | < 0.001 |  | 0.55 (0.31 – 0.73) | < 0.001 |  | 0.66 (0.45 – 0.80) | < 0.001 |
|  | ACC MVPA | 0.64 (0.42 – 0.79) | < 0.001 |  | 0.62 (0.39 – 0.77) | < 0.001 |  | 0.57 (0.33 – 0.74) | < 0.001 |  | 0.64 (0.44 – 0.79) | < 0.001 |
| Tuesday | ACC Total PA | 0.37 (0.09 – 0.60) | 0.009 |  | 0.18 (-0.11 – 0.45) | 0.21 |  | 0.32 (0.04 – 0.55) | 0.03 |  | 0.37 (0.10 – 0.59) | 0.009 |
|  | ACC MVPA | 0.26 (-0.03 – 0.51) | 0.07 |  | 0.20 (-0.10 – 0.46) | 0.17 |  | 0.22 (-0.06 – 0.48) | 0.12 |  | 0.26 (-0.02 – 0.51) | 0.07 |
| Wednesday | ACC Total PA | 0.43 (0.15 – 0.64) | 0.003 |  | 0.50 (0.23 – 0.69) | < 0.001 |  | 0.42 (0.15 – 0.63) | 0.004 |  | 0.42 (0.15 – 0.63) | 0.003 |
|  | ACC MVPA | 0.36 (0.07 – 0.59) | 0.01 |  | 0.50 (0.24 – 0.69) | < 0.001 |  | 0.36 (0.08 – 0.59) | 0.01 |  | 0.37 (0.09 – 0.59) | 0.011 |
| Thursday | ACC Total PA | 0.72 (0.54 – 0.84) | < 0.001 |  | 0.65 (0.45 – 0.80) | < 0.001 |  | 0.67 (0.48 – 0.80) | < 0.001 |  | 0.72 (0.55 – 0.84) | < 0.001 |
|  | ACC MVPA | 0.70 (0.51 – 0.82) | < 0.001 |  | 0.65 (0.44 – 0.79) | < 0.001 |  | 0.63 (0.42 – 0.77) | < 0.001 |  | 0.69 (0.51 – 0.82) | < 0.001 |
| Friday | ACC Total PA | 0.60 (0.36 – 0.76) | < 0.001 |  | 0.39 (0.11 – 0.62) | 0.007 |  | 0.58 (0.35 – 0.74) | < 0.001 |  | 0.60 (0.37 – 0.76) | < 0.001 |
|  | ACC MVPA | 0.52 (0.27 – 0.71) | < 0.001 |  | 0.37 (0.08 – 0.60) | 0.01 |  | 0.50 (0.24 – 0.69) | < 0.001 |  | 0.52 (0.28 – 0.71) | < 0.001 |
| Saturday | ACC Total PA | 0.54 (0.28 – 0.73) | < 0.001 |  | 0.38 (0.09 – 0.62) | 0.01 |  | 0.49 (0.22 – 0.69) | 0.001 |  | 0.56 (0.31 – 0.73) | < 0.001 |
|  | ACC MVPA | 0.45 (0.16 – 0.66) | 0.003 |  | 0.47 (0.19 – 0.68) | 0.002 |  | 0.38 (0.09 – 0.61) | 0.01 |  | 0.46 (0.19 – 0.67) | 0.002 |
| Sunday | ACC Total PA | 0.54 (0.26 – 0.73) | < 0.001 |  | 0.33 (0.02 – 0.59) | 0.03 |  | 0.46 (0.17 – 0.67) | 0.003 |  | 0.54 (0.27 – 0.72) | < 0.001 |
|  | ACC MVPA | 0.48 (0.19 – 0.69) | 0.002 |  | 0.39 (0.08 – 0.63) | 0.01 |  | 0.38 (0.09 – 0.62) | 0.01 |  | 0.48 (0.20 – 0.69) | 0.002 |
| Average/day | ACC Total PA^b^ | 0.44 (0.18 – 0.64) | 0.002 |  | 0.38 (0.11 – 0.59) | 0.007 |  | 0.41 (0.15 – 0.62) | 0.003 |  | 0.41 (0.15 – 0.62) | 0.002 |
|  | ACC MVPA^b^ | 0.29 (0.01 – 0.53) | 0.04 |  | 0.37 (0.10 – 0.58) | 0.009 |  | 0.29 (0.01 – 0.52) | 0.04 |  | 0.30 (0.02 – 0.53) | 0.03 |
|  | Aerobic capacity | -0.07 (-0.35 – 0.22)^a^ | 0.64 |  | -0.15 (-0.42 – 0.15) | 0.32 |  | -0.05 (-0.33 – 0.24) | 0.74 |  | 0.10 (-0.20 – 0.38) | 0.51 |

Notes: Spearman correlation coefficients are presented with 95% CI and p value based on either 46 (Monday), 49 (Tuesday), 47 (Wednesday), 48 (Thursday), 46 (Friday), 43 (Saturday), 41 (Sunday) or 50 (average per day) participants. ACC Total PA was defined as ≥ 50 mg, whereas ACC MVPA was defined as ≥ 100 mg. *ACC*Accelerometer*, CI* confidence interval, *MVPA* moderate-to-vigorous physical activity, *PA* physical activity, *PAQ24* Physical Activity Questionnaire for 24 h. ^a^ based on 49 participants due to reporting poor wellbeing during the submaximal test (results for male [*n* = 39] participants only: ρ = -0.10, 95% CI: -0.37 – 0.19, *p* = 0.59), ^b^ using weighted Spearman correlation coefficient.
