# Supplementary material for: Do we have to reduce the recall period? Validity of a daily physical activity questionnaire (PAQ24) in young active adults
Source: BMC Public Health. 2020 Jan 16;20:72. doi: 10.1186/s12889-020-8165-3 (PMC6966869; doi:10.1186/s12889-020-8165-3)
Supplement: Supplementary file 6 — Additional file 6. Relative agreement of VPA and ST between PAQ24 and accelerometer. [file 12889_2020_8165_MOESM6_ESM.docx]

**Additional file 6** Relative agreement of VPA and ST between PAQ24 and accelerometer

|  | **VPA** | ***p*** |  | **ST** | ***p*** |
| --- | --- | --- | --- | --- | --- |
| Monday | 0.49 (0.22 – 0.69) | < 0.001 |  | 0.47 (0.19 – 0.67)^a^ | 0.001 |
| Tuesday | 0.33 (0.05 – 0.57) | 0.02 |  | 0.22 (-0.07 – 0.48) | 0.12 |
| Wednesday | 0.45 (0.18 – 0.66) | 0.002 |  | 0.50 (0.24 – 0.69) | < 0.001 |
| Thursday | 0.33 (0.05 – 0.57) | 0.02 |  | 0.32 (0.04 – 0.56) | 0.02 |
| Friday | 0.14 (-0.16 – 0.42) | 0.34 |  | 0.58 (0.35 – 0.75) | < 0.001 |
| Saturday | 0.54 (0.27 – 0.72) | < 0.001 |  | 0.50 (0.23 – 0.70) | < 0.001 |
| Sunday | 0.57 (0.31 – 0.75) | < 0.001 |  | 0.22 (-0.10 – 0.50) | 0.16 |
| Average/day | 0.26 (-0.02 – 0.50) | 0.07 |  | 0.34 (0.07 – 0.57) | 0.02 |

Notes: Spearman correlations coefficients for each day and weighted Spearman correlation coefficient for average per day. Correlation coefficients are presented with 95% CI and p value based on either 46 (Monday), 49 (Tuesday), 47 (Wednesday), 48 (Thursday), 46 (Friday), 43 (Saturday), 41 (Sunday) or 50 (average per day) participants. The following comparison were made: PAQ24 VPA vs. ACC VPA, PAQ24 ST vs. ACC Inactivity. *ACC* Accelerometer*, CI* confidence interval, *PAQ24* Physical Activity Questionnaire for 24 h, *ST* sedentary time, *VPA* vigorous physical activity. ^a^ based on 45 participants due to four invalid days in the accelerometer assessment and another missing value in the PAQ24.
