# Supplementary material for: Do we have to reduce the recall period? Validity of a daily physical activity questionnaire (PAQ24) in young active adults
Source: BMC Public Health. 2020 Jan 16;20:72. doi: 10.1186/s12889-020-8165-3 (PMC6966869; doi:10.1186/s12889-020-8165-3)
Supplement: Supplementary file 7 — Additional file 7. Absolute agreement of Total PA, Total PA excl. Walking (MVPA), Total PA excl. Cycling and Total PA excl. Swimming between PAQ24 and different measures of the accelerometer. [file 12889_2020_8165_MOESM7_ESM.docx]

**Additional file 7** Absolute agreement of Total PA, Total PA excluding walking, Total PA excluding cycling and Total PA excluding swimming between PAQ24 and different measures of the accelerometer

|  |  | **Total PA** | **Total PA excluding walking** | **Total PA excluding cycling** | **Total PA excluding swimming** |
| --- | --- | --- | --- | --- | --- |
|  | Comparison  measure | M_diff_ ± 95% LOA | M_diff_ ± 95% LOA | M_diff_ ± 95% LOA | M_diff_ ± 95% LOA |
| Monday | ACC Total PA | 56 ± 222 | 166 ± 213 | 84 ± 237 | 59 ± 209 |
|  | ACC MVPA | -73 ± 235 | 37 ± 208 | -45 ± 235 | -70 ± 223 |
| Tuesday | ACC Total PA | 66 ± 233 | 173 ± 208 | 91 ± 244 | 67 ± 231 |
|  | ACC MVPA | -68 ± 232 | 39 ± 210 | -44 ± 201 | -67 ± 228 |
| Wednesday | ACC Total PA | 64 ± 286 | 203 ± 185 | 87 ± 286 | 66 ± 285 |
|  | ACC MVPA | -74 ± 295 | 69 ± 144 | -48 ± 295 | -69 ± 295 |
| Thursday | ACC Total PA | 56 ± 251 | 172 ± 207 | 76 ± 259 | 58 ± 251 |
|  | ACC MVPA | -79 ± 277 | 38 ± 210 | -59 ± 284 | -78 ± 278 |
| Friday | ACC Total PA | 50 ± 318 | 169 ± 272 | 68 ± 269 | 50 ± 268 |
|  | ACC MVPA | -95 ± 294 | 24 ± 275 | -77 ± 294 | -95 ± 294 |
| Saturday | ACC Total PA | 37 ± 313 | 185 ± 225 | 57 ± 314 | 38 ± 312 |
|  | ACC MVPA | -114 ± 333 | 35 ± 199 | -94 ± 325 | -113 ± 331 |
| Sunday | ACC Total PA | 87 ± 239 | 179 ± 199 | 102 ± 249 | 87 ± 237 |
|  | ACC MVPA | -46 ± 238 | 49 ± 167 | -30 ± 250 | -43 ± 238 |
| Average/day | ACC Total PA | 64 ± 180 | 64 ± 180 | 85 ± 185 | 65 ± 178 |
|  | ACC MVPA | -75 ± 183 | -75 ± 183 | -54 ± 186 | -74 ± 181 |

Notes: Results for ST and VPA are reported in the additional file 3. Absolute agreement by Bland-Altman analyses including mean difference and 95% limits of agreement (reported as minutes per day). Results are based on either 46 (Monday), 49 (Tuesday), 47 (Wednesday), 48 (Thursday), 46 (Friday), 43 (Saturday), 41 (Sunday) or 50 (average per day) participants. ACC Total PA was defined as ≥ 50 mg, whereas ACC MVPA was defined as ≥ 100 mg. *ACC* Accelerometer*, LOA* limits of agreement, *mg* milligravity units, *MVPA* moderate-to-vigorous physical activity*, PA* physical activity, *PAQ24* Physical Activity Questionnaire for 24 h, *ST* sedentary time, *VPA* vigorous physical activity.
